# Deletion of a Rare Fungal PKS CgPKS11 Promotes Chaetoglobosin A Biosynthesis, Yet Defers the Growth and Development of *Chaetomium globosum*

**DOI:** 10.3390/jof7090750

**Published:** 2021-09-13

**Authors:** Biyun Xiang, Xiaoran Hao, Qiaohong Xie, Guangya Shen, Yanjie Liu, Xudong Zhu

**Affiliations:** 1 Beijing Key Laboratory of Genetic Engineering Drug and Biotechnology, College of Life Sciences, Beijing Normal University, Beijing 100875, China; xby6024@126.com (B.X.); xqh1206@163.com (Q.X.); guangyashen@126.com (G.S.); yunher@bnu.edu.cn (Y.L.); 2 National Experimental Teaching Demonstrating Center, College of Life Sciences, Beijing Normal University, Beijing 100875, China; 3 Xiamen No.1 High School of Fujian, Xiamen 361000, China

**Keywords:** polyketide synthases, *Chaetomium globosum*, chaetoglobosin A, chaetoglocin A, sporulation, secondary metabolites

## Abstract

We previously reported that chaetoglobosin A (ChA) exhibits a great potential in the biocontrol of nematodes and pathogenic fungi. To improve the production of ChA, a CRISPR-Cas9 system was created and applied for eliminating potential competitive polyketide products. One of the polyketide synthase encoding genes, *Cgpks11*, which is putatively involved in the biosynthesis of chaetoglocin A, was disrupted. *Cgpks11* deletion led to the overexpression of the *CgcheA* gene cluster, which is responsible for ChA biosynthesis, and a 1.6-fold increase of ChA. Transcription of *pks-1*, a melanin PKS, was simultaneously upregulated. Conversely, the transcription of genes for chaetoglocin A biosynthesis, e.g., CHGG_10646 and CHGG_10649, were significantly downregulated. The deletion also led to growth retardation and seriously impaired ascospore development. This study found a novel regulatory means on the biosynthesis of ChA by CgPKS11. CgPKS11 affects chaetoglobosin A biosynthesis, growth, and development in *Chaetomium globosum*.

## 1. Introduction

Owing to diverse bioactivities, small molecule secondary metabolites have been major sources of medicines, promising pesticides, contaminants of food, biological probes, and targets of study for synthetic and analytical chemists [1,2]. Polyketides encompass a highly structurally diverse group of secondary products. Chemical skeletons of polyketides are assembled through sequential additions of two carbon building blocks derived from acetyl-coenzyme A (CoA) or malonyl-CoA precursors by polyketide synthases (PKSs) [3,4]. Based on the structural organization of their functional domains, PKSs are classified into three basic categories: type I PKSs are large multifunctional proteins comprised of several functional domains and found in both bacteria and fungi; type II PKSs are formed by discrete catalytic domains and are typically found in bacteria; type III PKSs are simpler chalcone synthase-type enzymes that catalyze the formation of the product within a single active site, mainly in plants and bacteria. Type I PKSs can be further classified into single modular iterative type I PKS and multi-modular PKS. In the fungi kingdom, the iterative type I PKS is more often encountered. In some cases, modules from type I PKSs are linked to non-ribosomal peptide synthetase (NRPS) modules, which results in the production of polyketide-peptide hybrid metabolites, e.g., cytochalasans [3,5,6,7].

**Figure 1:**
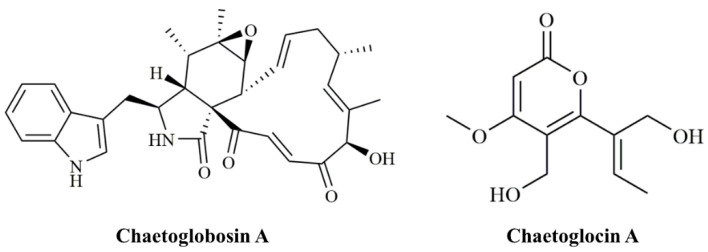
Chemical structures of chaetoglobosin A and chaetoglocin A.

Chaetoglobosin A (ChA, Figure 1) was initially identified in *Chaetomium globosum* [8]. Structurally, ChA contains a 10-(indol-3-yl) group and a tricyclic core in which a macrocyclic ring is fused to a perhydroisoindolone moiety [9]. Like most cytochalasins, ChA has been confirmed to target filamentous actin in mammalian cells, and thereby induces cell-cycle arrest and inhibits membrane ruffling and cell migration [10], endowing ChA strong cytotoxicity against tumor cell lines, immunomodulatory activities, and antifungal activities [11,12,13]. Even more, we previously reported the nematicidal activity of culture filtrates and ChA from *C. globosum* NK102 against the nematode *Meloidogyne incognita*. Our results showed that both filtrates and purified ChA demonstrated strong adverse effects on the second stage juveniles (J2s) mortality with 99.8% at 300 μg/mL (LC_50_= 77.0 μg/mL) [14]. Later, Ashrafi extracted ChA from the fermentation broth of the destructive parasitic fungus of the cereal cyst nematode *Heterodera filipjevi* and demonstrated that ChA caused a temporary inhibition on J2 mobilization [15]. Thus, it is intriguing to explore the nematicidal activity of *C. globosum*, and its use as a biological agent for the control of plant pathogenic microorganisms and pests.

The genetic and molecular basis of ChA biosynthesis in fungi was initially studied in *Penicillium expansum* [16], in which genes involved in ChA biosynthesis are clustered, and the carbon scaffold of ChA is synthesized by a hybrid iterative type I polyketide synthase-non-ribosomal peptide synthetase (PKS-NRPS) CheA and a standalone enoyl reductase CheB. After a spontaneous intramolecular condensation and a Diels–Alder reaction, the key intermediate prochaetoglobosin is generated, which can be furnished into ChA after a set of oxidative modifications. Through gene disruption, homologs of CheA (*CgcheA* with a gene locus CHGG_01239) and CheB (*CgcheB* with a gene locus CHGG_01238) as well as the enzymes CheD (*CgcheE* with a gene locus CHGG_01242-1), CheE (*CgcheF* with a gene locus CHGG_01242-2), and CheG (*CgcheG* with a gene locus CHGG_01243), involved in the final oxidative transformations, are identified and characterized in *C. globosum* [17,18]. Nonetheless, we previously observed in *C. globosum* NK102 that a putative pigment polyketide synthase gene, *pks-1/alb1*, was also required for ChA biosynthesis [19]. The knock-down of *pks-1* resulted in a dramatic reduction of ChA production and significant inhibition of pigmentation and sporulation. It is proposed that PKS-1 probably provides precursors for ChA biosynthesis.

The involvement of *pks-1* in the production of ChA prompts us to the possibility that biosynthesis of polyketides in fungi may have competition for precursors between PKSs. Thus, we conducted bioinformatics analysis using the software antiSMASH (https://antismash.secondarymetabolites.org, accessed on 17 June 2019) and protein Blast against the NCBI database and identified 28 predicted PKS genes in the genome of *C. globosum* (Table S1). In order to eliminate potential competing polyketide products, we tried to delete 26 putative PKS encoding genes, except for *CgcheA* and *pks-1*, respectively. A previously reported, PKS encoding ORF in another *C. globosum* strain, CHGG_10647, on scaffold_7 was predicted to play a role in the biosynthesis of chaetoglocin A (Figure 1) [20]. We confirmed its role in the production of chaetoglocin A. We deleted the homologous gene of CHGG_10647 (named as *Cgpks11*) in *C. globosum* NK102 via a “suicide” CRISPR-Cas9 system. Interestingly, we found that the deletion of *Cgpks11* leads to a significant increase of ChA production, and yet this dramatically delayed the growth of mycelium, sporulation, and sexual development. We also discuss this new phenomenon as to the role of this gene and its derived polyketide during ChA biosynthesis.

## 2. Materials and Methods

### 2.1. Strains and Growth Conditions

The wild-type strain *C. globosum* NK102, which produces a high level of ChA, was isolated and stocked by our laboratory, and was the host strain for gene knockout in this study. NK102 was grown on potato dextrose agar (PDA) at 28 °C. For preparation of protoplasts, approximately (1–4) ×10^4^ ascospores, collected from a single plate, were used to inoculate 100 mL of potato dextrose broth (PDB) medium, shaken at 28 °C and 200 rpm for 2 days. Ten grown mycelial balls were transferred into 100 mL fresh PDB medium, and then continued to grow for 3 days at 28 °C with a shaking speed of 160 rpm. For DNA isolation, 5 mm agar plaques containing the fungal hyphae were inoculated in 100 mL PDB and incubated for 3 days in a rotary shaker at 28 °C and 200 rpm. For high-performance liquid chromatography (HPLC) analysis or RNA isolation, strains were cultured in 100 mL PDB for 7 days at 28 °C and shaken at 200 rpm. *Escherichia coli*, used for constructing plasmids and conjugation, was grown in Luria–Bertani (LB) broth (Difco, Detroit, MI, USA) or on LB agar plates at 37 °C.

### 2.2. Gene Prediction, Alignment, and Phylogenetic Analysis

The PKS gene clusters in *C. globosum* CBS148.51 genome (RefSeq: NZ_AAFU00000000.1) were analyzed by using the antiSMASH (https://antismash.secondarymetabolites.org, accessed on 17 June 2019) [21]. Peptide sequences of all predicted genes were then submitted to protein BLAST against the NCBI database for further confirmation.

The homologous gene of CHGG_10647 in *C. globosum* NK102, subsequently named *Cgpks11*, was obtained by protein BLAST. The putative amino acid sequence of *Cgpks11* was then subjected to protein BLAST against the NCBI database for more information. The putative conserved domains of CgPKS11 were defined from the NCBI Conserved Domains database and further confirmed by searching on the UniProtKB database. For the phylogenetic evaluation of CgPKS11, all hits producing E-values below 10^−40^ generated from protein BLAST were taken into subsequent analysis. Hypothetical protein, uncharacterized protein, and repetitive sequences of the same functional proteins in the same strain were removed. The full-length predicted amino acid sequences of CgPKS11 and the selected well-characterized proteins were aligned using the Muscle program in MEGA Ⅹ (v10.1.8), and the alignment was used to generate a phylogenetic tree by using the Neighbor-Joining algorithm and Jones–Thornton–Taylor (JTT) matrix-based model. The alignment gaps and missing data sites were deleted, and a bootstrap value based on 1000 replications was used to measure statistical confidence of branch nodes of the phylogenetic tree.

### 2.3. Construction of CRISPR-Cas9 Editing Tool in C. globosum

Previously, we reported a “suicide” CRISPR-Cas9 system to promote gene deletion with a minimized off-target effect by eliminating the system itself upon the occurrence of recombination and a suitable system for gene restoration assay in *Cryptococcus neoformans* [22]. In this study, a similar system was constructed in *C. globosum* NK102. Plasmids for gene deletion were constructed based on recombination cloning method using In-Fusion^®^ HD Cloning kits (Takara Bio, Mountain View, CA, USA). To construct the Cas9 expression cassette, a Cas9 coding region with NLSs followed by a bGHpA terminator was amplified from pBS-URA5-Pact: Cas9 [22] by primers Cas9in-F/Cas9in-R. An 800 bp ACTIN promoter was amplified from *C. globosum* NK102 genomic DNA with primers act1p-F/act1p-R. Vector pUCATPH [23] was linearized by restriction enzyme *Hind* III. Primer act1p-R and Cas9in-F shared 15 homologous bases at each 5′-terminal. Primers act1p-F and Cas9in-R shared 15 homologous bases with the cohesive terminus of linearized pUCATPH, respectively. The amplicons of the expected sizes were purified with the GeneJET Gel Extraction Kit (Thermo Fisher Scientific, Carlsbad, CA, USA) and quantified using NanoDropTM One Spectrophotometer (Thermo Fisher Scientific, Madison, WI, USA). To fuse the ACTIN promoter and CAS9 coding region into the *Hind* III site of pUCATPH to generate pUCATPH-Cas9, the In-Fusion reaction was performed in a total volume of 5 μL, containing 1.0 μL of 5× In-Fusion HD Enzyme Premix, 100 ng of each purified PCR fragment, and ddH_2_O from the In-Fusion HD PCR Cloning Kit. The reaction mix was incubated at 50 °C for 15 min, and then placed on ice for transformation using *E. coli* DH5α competent cells. Transformants were screened with colony PCR using primers act1p-F and CXcas9infu-1. To construct the single guide DNA (gDNA) expression cassette for the production of the single gRNA, a 200 bp U6 promoter was amplified from *C. globosum* NK102 genomic DNA with primers u6gdna1-F and u6gdna1-R. A bacterial tracerRNA scaffold was amplified from plasmid pBS-URA5-Pact: Cas9 with primers u6gdna2-F and u6gdna2-R. Vector pUCATPH-Cas9 was linearized by restriction enzyme *Kpn* I. In order to reuse the plasmid to target other genes, two *Bbs* I restriction sites were designed at the 5′-terminal of the primers u6gdna1-R and u6gdna2-F for insertion of the target sequence GN_19_NGG [22]. primers u6gdna1-F and u6gdna2-R shared 15 homologous bases with the cohesive terminus of linearized pUCATPH-Cas9, respectively. As described above, the amplicons of the expected sizes were purified, quantified, and fused into the *Kpn* Ⅰ site of pUCATPH-Cas9 to generate the plasmid pCgCRISPR. The transformants were screened with colony PCR using the primers u6gdna1-F and u6gdna2-R.

In the CRISPR-Cas9 editing system, a GN_19_NGG guide sequence was designed with the following parameters:

(1) The sequence was located close to the 5′ end of the target gene-coding region for cleavage;
(2) Similarity to the GN_19_NGG was searched by BLASTN to the *C. globosum* genome to avoid cleavage at secondary loci.

### 2.4. CRISPR-Cas9 Mediated Cgpks11 Deletion

To construct the *Cgpks11* knockout vector, the *Hyg^R^* cassette flanked by *Xho* I and *Xba* I sites and amplified from pUCATPH by primers Hyg- *Xho* I -F and Hyg- *Xho* I -R, was introduced into the *Xho* I site of plasmid pCgCRISPR, and the resulting plasmid was named pCRISPR-Hyg. Then, the vector pCRISPR-Hyg was linearized by the restriction of enzyme *Bbs* I. The guide GN_19_NGG sequence to *Cgpks11* was designed for designing the complementary primers PCgpks11-N19-F and PCgpks11-N19-R. The cohesive terminus of *Bbs* I was added at the 5′ terminal of each primer, respectively. These primers were synthesized, denatured (95 °C, 10 min), and then annealed at room temperature to generate the GN_19_NGG fragment. The GN_19_NGG fragment was ligated into the *Bbs* I site of pCRISPR-Hyg to generate plasmid PCgpks11-N19. The upstream (877 bp) and downstream (938 bp) flanking sequences of the *Cgpks11* were amplified from NK102 genomic DNA with primers PCgpks11-UF/PCgpks11-UR and PCgpks11-DF/PCgpks11-DR, respectively. PCgpks11-N19 was cut by *Xba* I to generate a large fragment containing Cas9 and a small fragment containing a *Hyg^R^* cassette bearing the hygromycin B resistance as a selection marker. The 5′ and 3′ flanks of *Cgpks11* and the *Hyg^R^* cassette were fused with the largest fragment via In-Fusion HD Cloning kits, as mentioned above (Figure S1). The fused plasmid, designated as pCgpks11, was linearized with *Xba* I and introduced into the NK102 via PEG-mediated protoplast transformation. All primers used in this assay are listed in Table S2.

### 2.5. Fungal Transformation and Transformants Screening

Protoplasts were prepared and transformed as described [24]. For transformants selection, hygromycin B (Sigma-Aldrich, St. Louis, MO, USA) was added to the plates at a final concentration of 100 μg/mL.

In screening for Δ*cgpks11* mutants, two PCR reactions per transformant were carried out. Mutants were subjected to PCR analysis using a combination of a marker-specific primer and a primer designed to anneal the outside of the homologous region flanking the *Hyg^R^* gene. Primers P11-KO-VP-F/iHYG-R and primers P11-KO-VP-R/PHYG-ter-F could amplify fragments of 1550 bp and 1367 bp, respectively. For further confirmation, genomic DNA was extracted and subjected to Southern blot analysis as previously described [24]. A 2114 bp *Hyg^R^* cassette, amplified from pUCATPH using primers HYG-F/HYG-R, was labeled as the probe. Experiments involving DNA labeling, hybridization, and detection were carried out according to the instructions of the DIG High Prime DNA Labeling and Detection Starter Kit II (Roche Life Science, Mannheim, Germany). All primers were listed in Table S2.

### 2.6. RNA Preparation and Quantitative Real-Time PCR

Total RNA was extracted from the lyophilized and ground mycelium using an RNAiso Plus kit (Takara Bio, Shiga, Japan). The first-strand cDNA was generated by reverse transcription in a 20 μL reaction using the TransScript First-Strand cDNA Synthesis SuperMix kit (TransGen Biotech, Beijing, China). Quantitative real-time PCR was performed by LightCycler^®^ 480 instrument III (Roche Diagnostics, Indianapolis, IN, USA). Each reaction of 20 μL PCR was performed with SYBR Green I Master (Roche Life Science, Mannheim, Germany). Reactions were set up in three replicates per sample. Controls without the addition of the templates were included for each primer set. PCR cycling parameters were pre-incubation at 95 °C for 5 min, followed by 45 cycles of denaturation at 95 °C for 10 s, annealing at 55 °C for 15 s, and extension at 72 °C for 20 s. The qRT-PCR data was analyzed using the $2^{-\Delta \Delta \mathrm{CT}}$ relative quantification method [25] to calculate the relative expression levels of genes. The housekeeping gene encoding glyceraldehyde-3-phosphate dehydrogenase (GAPDH) was used as reference. The amplification efficiencies of the target and reference genes were compared at different template concentrations. The gene-specific pairs of primers used in the amplifications were as follows: qCgpks11F/qCgpks11-R for *Cgpks11*, q10646-F/q10646-R for CHGG_10646, q10649-F/q10649-R for CHGG_10649, q10650-F/q10650-R for CHGG_10650, q01237-F/q01237-R for CHGG_01237, q01238-F/q01238-R for CHGG_01238, q01239-F/q01239-R for *CgcheA*, q01240-F/q01240-R for CHGG_01240, q01241-F/q01241-R for CHGG_01241, q01242-1-F/q01242-1-R for CHGG_01242-1, q01242-2-F/q01242-2-R for CHGG_01242-2, q01243-F/q01243-R for CHGG_01243, q01244-F/q01244-R for CHGG_01244, q00542-F/q00542-R for *pks-1*, and qGAPDH-F/qGAPDH-R for GAPDH gene (Table S2).

### 2.7. Growth Observation

The growth of Δ*cgpks11* mutants was compared with the WT strain on agar plates and in liquid medium. The linear growth rate of mutants on PDA media at 28 °C was determined in three replicate plates (D = 9 cm) by measuring the diameter of the colony every day over a 7-day period. For the submerged culture, the total mycelium of each strain was isolated from cultures inoculated in 100 mL PDB medium for 7 days at 28 °C, with shaking at 200 rpm, respectively. Fresh mycelium was isolated by vacuum pump and dry mycelium was obtained by vacuum freeze-drying. The weight of the dry mycelium was determined.

### 2.8. Quantification of Ascospores Production

Ascospores were harvested from 15-day-old cultures on PDA at 28 °C in triplicate for each strain. Firstly, 2 mL of sterile distilled water was added to each plate. Then, ascospores formed on each plate were scrapped using a sterilized spatula and was resuspended in 30 mL of sterile distilled water. After centrifugation at 3000× *g* for 10 min, the supernatant full of hyphae-debris was discarded, and the ascospores pellet was washed twice with sterile distilled water and finally resuspended in 10 mL sterile distilled water. The concentration of ascospores suspension in sterile distilled water was determined by hemocytometry under the Moticam Ⅹ3 microscope (Motic, Xiamen, China).

### 2.9. Measurement of the Cell Length of Ascoma Hairs

Ascocarps were scrapped from a 15-day-old culture and observed using a 40×/0.65NA objective lens on a Moticam X3 microscope. MotiConnect software was used to measure the cell length of ascoma hairs and photographs. Ten ascocarps and two mature septate ascoma hairs for each ascocarp were measured.

### 2.10. Microscopy

Agar plaques containing the fungal hyphae of Δ*cgpks11*, or the WT strain were inoculated on PDA plates and cultured at 28 °C. Perithecia that formed on the plates was scrapped off. To observe the asci and ascospores that developed within a perithecium, we gently tapped the cover slip with the holder of the inoculating loop and crack the perithecium. Images were taken by using 10×/0.25NA and 40×/0.65NA objective lenses on a Moticam Ⅹ3 microscope. For microcopy observation of mature ascospores, a Carl Zeiss Axio Imager Z2 Apotome2 Upright Microscope (Carl Zeiss, Oberkochen, Germany) equipped with EC P1an-NEOFLUAR 40×/0.75NA and Plan-APOCHROMAT 63×/1.40NA objective lenses (Carl Zeiss, Göttingen, Germany) were used. Images were captured with an Axiocam 506 mono microscopy camera (Carl Zeiss, Göttingen, Germany) and processed using ZEN 2.3 lite software.

### 2.11. Detection of Chaetoglobosin A by HPLC

After 7 days of cultivation, the fermentation cultures of the WT and Δ*cgpks11* were passed through a Buchner funnel vacuum filter, and the filter residue, i.e., mycelial pellets were subjected to lyophilized and weighed, respectively. Each sample of the filtrate was added in an equal volume of ethyl acetate and stood for 12 h after agitation to extract ChA through layering. The organic phase was then concentrated, dissolved, and centrifuged as previously reported [24]. The supernatant was filtered through a 0.22 μm Millipore filter and subjected to HPLC analysis on Agilent 1200 HPLC system (Agilent Technologies, Santa Clara, CA, USA) with Kromasil C18 ODS column (4.6 × 250 mm, AKZO Nobel, Gland, Switzerland). The UV detection wavelength was set at 227 nm, and the sample flow rate was set at 1 mL/min. Standard ChA (Sigma-Aldrich, St. Louis, MO, USA) served as the control. For quantification of ChA, a standard curve was created with known concentrations of the standard sample.

## 3. Results

### 3.1. Sequence and Phylogenetic Analyses of CgPKS11

**Figure 2:**
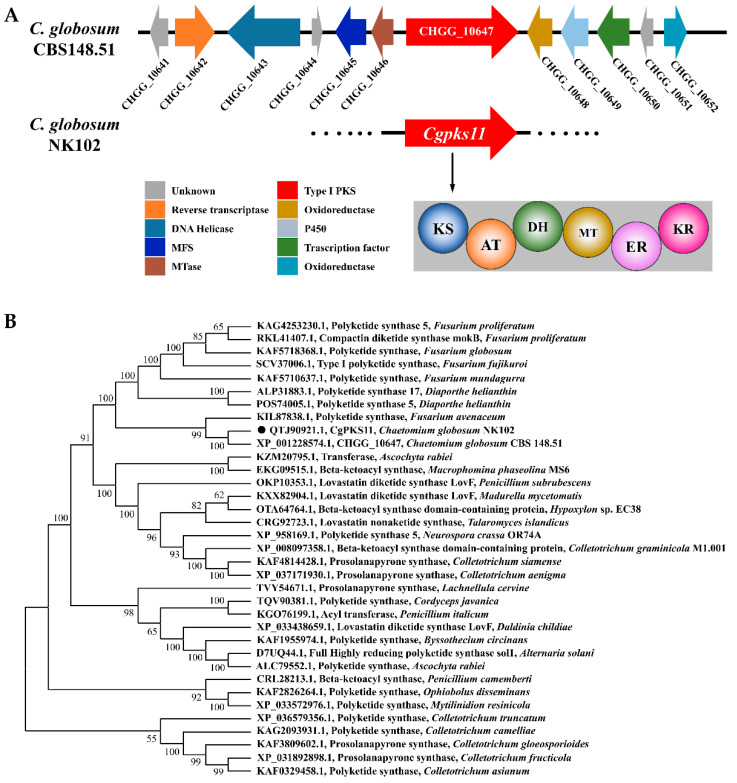
Prediction and phylogenetic analysis of the CgPKS11 in *C. globosum*. (**A**) The organization of the chaetoglocin A biosynthetic gene cluster in *C. globosum*. The predicted function of genes in this cluster are shown by different colors. The predicted functional domains of CgPKS11 are represented by colored circles: KS = ketosynthase, AT = acyltransferase, DH = dehydratase, MT = methyltransferase, ER = enoyl reductase, and KR = ketoreductase. (**B**) A phylogenetic tree based on the full-length amino acid sequences of CgPKS11 and another 34 homologous PKSs. Phylogeny was inferred by the NJ method implemented in MEGA Ⅹ under the JTT matrix-based method. Bootstrap supports > 50% from 1000 replicates per run are labeled on branch nodes. Polyketide synthases in *Colletotrichum* spp. were designated as the outgroup. CgPKS11 from the *C. globosum* NK102 clade was marked in black dot.

The AntiSMASH analysis of the model strain *C. globosum* CBS148.51 genome indicated that CHGG_10647 was located in a biosynthetic gene cluster, and the predicted functional genes of this cluster are shown in Figure 2A. The homologues gene of CHGG_10647 in *C. globosum* NK102 was designated as *Cgpks11* (GenBank accession no. MW815637). The deduced CgPKS11 protein is composed of 2581 amino acids with 3 exons. The amino acid sequence of CgPKS11 shares 95.82% identity with CHGG_10647. NCBI’s Conserved Domain database predicted that the putative CgPKS11 protein has the following domain organization: KS-AT-DH-MT-ER-KR, which is characteristic of type I polyketide synthase (Figure 2A). CgPKS11 contains the KS and AT domains that are essential for constructing the product chain of polyketides. It is likely to be highly reduced, since it contains all three reducing domains (ER, KR, and DH domains) required for the stepwise reduction of the substrate. CgPKS11 contains a MT domain that is the most likely for methylation of the substrate. Unlike typical type I PKSs, CgPKS11 lacks the acyl carrier protein (ACP) domain that is essential for polyketides chain elongation and chain modification on the bound acyl chain [26].

For more information to predict the function of CgPKS11, the amino acid sequence of CgPKS11 was subjected to protein BLAST against NCBI database. Overall, 34 well-characterized homologs (>40% identity) of CgPKS11 were selected. The full-length predicted amino acid sequence of CgPKS11 and the 28 homologs were aligned using the program Muscle. With the Neighbor-Joining method on MEGA Ⅹ (v10.1.8), a phylogenetic tree with these known PKSs was constructed (Figure 2B). This analysis suggests that CgPKS11 is in the same clade with a polyketide synthase from *Fusarium avenaceum* and the percentage of replication reached 99% in bootstrap test, and these two polyketide synthases have 56.6% similarity when aligned using protein BLAST. In addition, polyketide synthase 5 and polyketide synthase 17 from *Diaporthe helianthi*, as well as type I polyketide synthase from *F. fujilkuroi,* were characterized as PKSs that share high identity with CgPKS11.

### 3.2. Targeted Deletion of Cgpks11 with the CRISPR-Cas9 System

**Figure 3:**
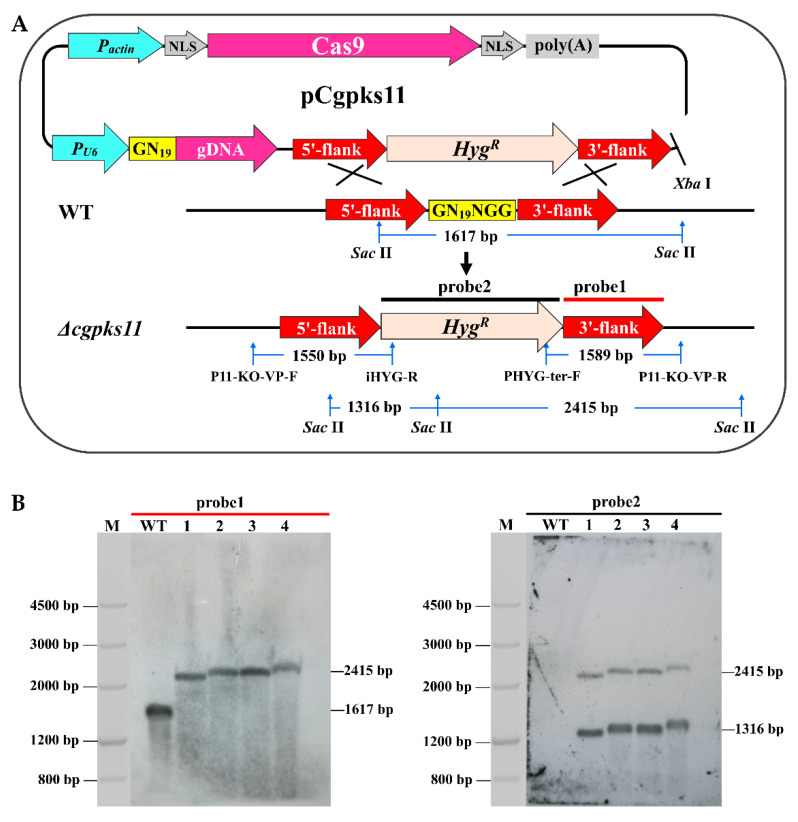
Targeted deletion of *Cgpks11* in *C. globosum* via a “suicide” CRISPR/Cas9 system. (**A**) Diagram of the targeted deletion of *Cgpks11* via the CRISPR/Cas9-mediated homologous recombination. (**B**) Southern blot analysis of Δ*cgpks11* mutants. M: Marker III. Lanes 1 to 4 were loaded samples of transformants NO11, NO12, NO15, and NO16, respectively.

CgPKS11 deletion was conducted via a “suicide” CRISPR-Cas9 system as previously reported [22]. Plasmid constructed for *Cgpks11* deletion was named pCgpks11 (Figure 3A), linearized, and introduced into *C. globosum* NK102 via PEG-mediated protoplast transformation. Nine transformants were analyzed by diagnostic PCR amplification and sequenced with two pairs of primers. Transformants that underwent a correct recombination event would produce amplicons of a desired size, e.g., a 1550 bp fragment could be amplified by primers P11-KO-VP-F/iHYG-R, and a 1367 bp fragment could be amplified by primers PHYG-ter-F/P11-KO-VP-R. As shown in Figure S2, four transformants acquired the anticipated fragments, which suggested that correct gene deletion occurred at the target locus in these transformants.

Southern blotting was performed as a further verification of the transformants (Figure 3B). Genomic DNA of these four transformants, NO11, NO12, NO15, and NO16, was prepared and digested by *Sac* II. There was one *Sac* II site located in the hygromycin B resistance cassette and two *Sac* II sites located in the *Cgpks11* gene cassette, one located in the 5′-flank, and another lied outside of the 3′-flank (Figure 3A). When probed with a 938 bp 3′-flank of *Cgpks11* (named probe1, highlighted in red), a 2415 bp band containing part of the sequence of *Hyg^R^* should be detected in the mutants, while a shorter 1617 bp band should be detected in the WT strain. All transformants were proved positive, ascertaining that the native copy of *Cgpks11* was disrupted in the transformants as anticipated.

To determine whether there were other random insertion events. The probe1 was washed away and the nylon membrane was detected with probe2, a labelled 2114 bp *Hyg^R^* cassette. As shown in Figure 3B, there were two anticipated bands in each lane of Δ*cgpks11* transformants, while no signal was detected in the lane of the WT. These results further confirmed that a single copy of the interference cassette was inserted into the genome of these transformants.

In order to ensure that the residual Cas9 cassette was re-excised, another Southern blot was conducted. Genomes of transformants mentioned above as well as another five random selected transformants were extracted and digested by *EcoR* I. As shown in Figure S3, the Cas9 expression cassette was successfully eliminated in transformants NO11, NO12, NO15, NO16, and NO23, while one or more copies of Cas9 expression cassette was inserted in genomes of transformants NO1, NO3, NO9, and NO33. These results suggest that the elimination of Cas9 succeeded and *Cgpks11* was deleted as anticipated. Transformant NO12, named Δ*cgpks11*, was selected for subsequent analysis.

### 3.3. CgPKS11 Deletion Increased the Production of ChA

**Figure 4:**
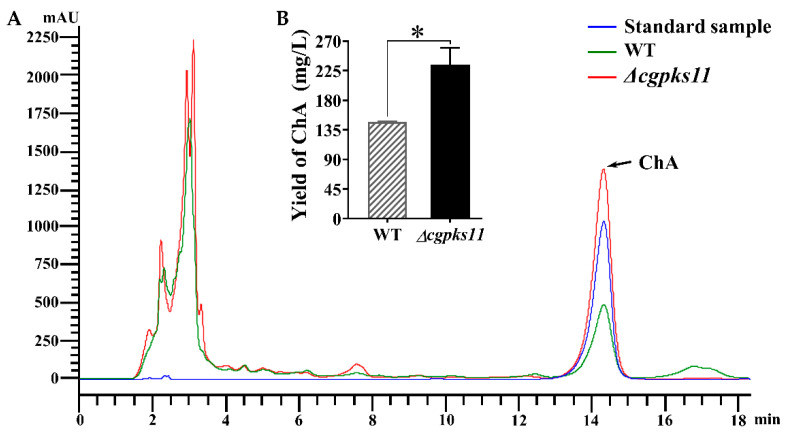
CgPKS11 deletion led to increased production of ChA. (**A**) HPLC analysis on the production of ChA in the WT and Δ*cgpks11*. (**B**) Yields of ChA in the WT and Δ*cgpks11*. Three culture replicates of each strain were used. There is significantly difference between the mutant and wild-type strains, as indicated by an asterisk (*p*-value < 0.01 with *t*-test analysis).

Our purpose of the study was to determine the effect of eliminating CgPKS11 on ChA biosynthesis. Thus, the yield of ChA from Δ*cgpks11* was compared with that from the WT strain. Growth conditions for ChA biosynthesis were initially determined to be 100 mL PDB at 28 °C for 7 days. HPLC was employed for ChA detection and quantitation. Like the WT strain, mutant Δ*cgpks11* had a characteristic peak of ChA at the retention 14.431 min (Figure 4A). However, the concentration of ChA in the Δ*cgpks11* was significantly increased to 234 mg/L, about 1.6-fold of that in the WT strain (146 mg/L), as is shown in Figure 4B.

### 3.4. CgPKS11 Deletion Resulted in Slow Growth and Retarded Sporulation

**Figure 5:**
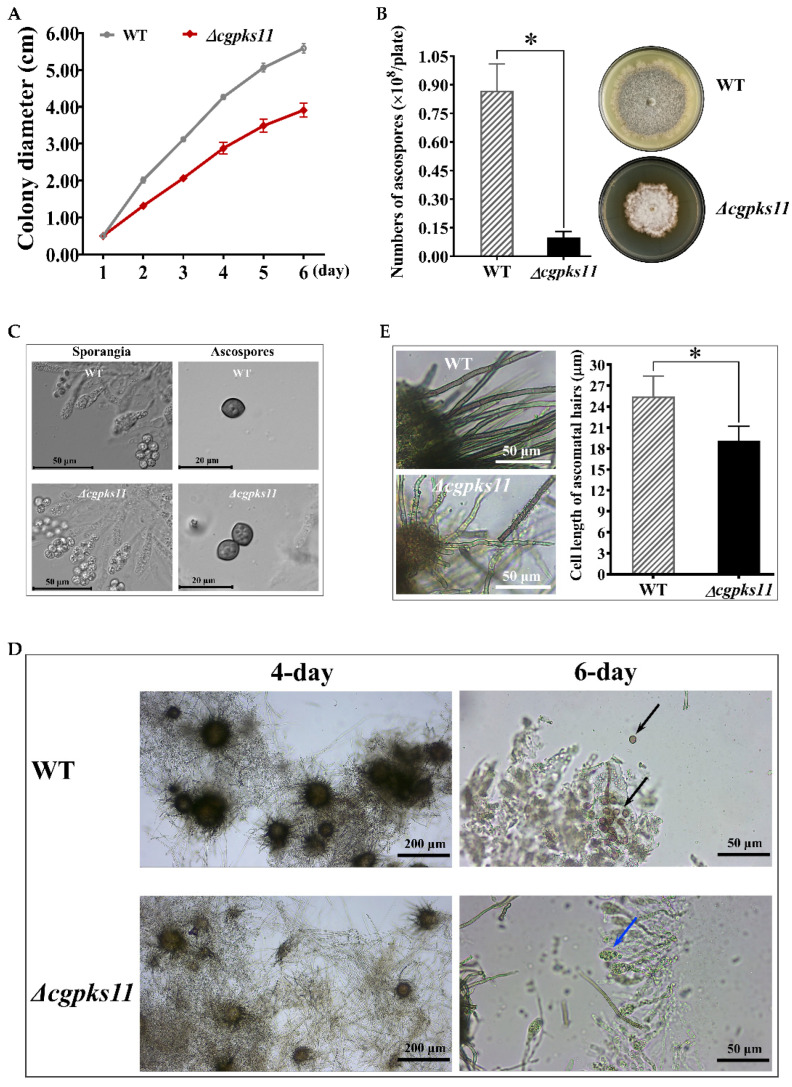
CgPKS11 deletion led to decelerated growth and delayed sporulation. (**A**) The growth curve of WT and Δ*cgpks11* strains when cultivated in PDA plates. The Δ*cgpks11* mutant was inoculated in PDA plate supplemented with 100 μg/mL hygromycin B. (**B**) Comparison of Δ*cgpks11* with WT on colony morphology and sporulation ability. (**C**) Morphology characteristics of sporangia and ascospores produced from WT and Δ*cgpks11* mutant. (**D**) Maturation of ascospores in WT and Δ*cgpks11* strains. Left (ascocarps), under the 10× objective lens; right, the black and blue arrow indicated the mature ascospores and immature ascospores in sporangium under the 40× objective lens, respectively. (**E**) Cell length of ascoma hairs from WT and Δ*cgpks11* strains was observed and measured under the microscope by using the 40× objective lens. There is significantly difference between the mutant and WT as indicated by an asterisk (*p*-value < 0.01 with *t*-test analysis).

We observed the growth of the Δ*cgpks11* on agar and in liquid. During a 7-day submerged culture, the mycelial growth rate as well as the pigment production of the Δ*cgpks11* and the WT did not show a remarkable difference (Figure S4). However, when cultured on agar plates (Figure 5A), the mycelial growth rate of the Δ*cgpks11* mutant was significantly lower than that of the WT strain, e.g., the diameter of a 6-day colony of WT strain reached 6 cm, while that of Δ*cgpks11* just approached 4 cm.

In addition, the WT strain produced extremely dense ascocarps and its aerial mycelia grew robustly. By contrast, the Δ*cgpks11* mutant exhibited albino mycelium with sparse ascocarps. Subsequent qualification of ascospores further confirmed that deletion of *Cgpks11* affected the sexual development of *C. globosum*. Analysis of 15-day-old cultures on PDA medium revealed that the ascospores yields from the WT and Δ*cgpks11* strains were 0.87 ± 0.14 × 10^8^ (*p* < 0.01) and 0.10 ± 0.03 × 10^8^ (*p* < 0.01) ascospores per plate, respectively, the yields of the mutant being decreased by 96.0% relative to WT (Figure 5B). However, deletion of *Cgpks11* has no noticeable effect on the amount and morphology of ascospores in a sporangium. The sporangia from the WT strain or from the Δ*cgpks11* uniformly contained eight lemon-shaped ascospores (Figure 5C).

We then detected the maturation of ascocarp. As shown in Figure 5D, the WT strain produced a large amount of fuscous ascocarp after 4 days, while ascocarp formed from the Δ*cgpks11* were significantly smaller and light colored. When cultured for 6 days, we could observe mature ascospores released from open perithecium, while ascospores from the Δ*cgpks11* remained little changed in ascocarp. The maturation time of ascocarp from the Δ*cgpks11* was delayed to approximately 8 days.

Deletion of *Cgpks11* also led to slightly shortened cells of the ascoma hairs. The average cell length of ascoma hairs of the WT and Δ*cgpks11* were 25.46 ± 2.91 μm and 19.12 ± 2.07 μm, respectively. Thus, the cells of ascoma hairs from the Δ*cgpks11* mutant were shorter than those of the WT strain (Figure 5E).

### 3.5. Increased Transcription of ChA Biosynthesis Gene Cluster and pks-1 in the Δcgpks11 Mutant

**Figure 6:**
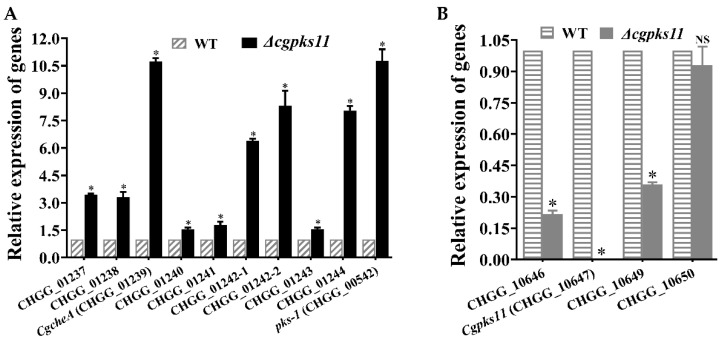
Expression of genes involved in ChA and chaetoglocin A biosynthesis in the wild-type strain and Δ*cgpks11* mutant. (**A**) The expression of the ChA biosynthesis gene cluster (genes from CHGG_01237 to CHGG_01244) and *pks-1* that were proved to be associated with ChA biosynthesis. (**B**) Detection of transcription levels of the genes involved in chaetoglocin A biosynthesis. There is significantly difference between the mutant and wild-type strains as indicated by an asterisk (*p*-value < 0.01 with *t*-test analysis). Experiments were performed in triplicate.

Expression of genes involved in ChA biosynthesis in the Δ*cgpks11* mutant was assessed with qRT-PCR. Genes located in the ChA biosynthesis gene cluster were simultaneously increased in the Δ*cgpks11* mutant (Figure 6A). The mRNA level of the ChA carbon scaffold biosynthesis gene *CgcheA* in Δ*cgpks11* mutant was significantly increased to 10.7-fold of that in the wild-type strain. The mRNA levels of the transcription factor encoding gene CHGG_01237, the enoyl reductase encoding gene CHGG_01238, and three redox enzymes encoding genes CHGG_01242-1, CHGG_01242-2, and CHGG_01243 in the Δ*cgpks11* mutant were increased to 3.4-fold, 3.3-fold, 6.4-fold, 8.3-fold, and 1.6-fold, respectively, of that in the WT. Previously, we reported that spore pigment polyketide synthase PKS-1 was also involved in ChA biosynthesis and silenced *pks-1* resulted in downregulation of ChA biosynthesis. Thus, the expression of *pks-1* in the Δ*cgpks11* mutant was also examined. The mRNA level of *pks-1* in Δ*cgpks11* was also sharply increased to 10.7-fold of that in the wild-type strain as *CgcheA*. This qRT-PCR result explained why the ChA yield was increased, although it is difficult to find a co-relationship between the *Cgpks11* and the ChA biosynthesis.

### 3.6. Effects of the Cgpks11 Deletion on Genes in Chaetoglocin Biosynthesis Cluster

Expression of other genes within the chaetoglocin A biosynthesis gene cluster was examined by qRT-PCR in the mutant Δ*cgpks11*. As shown in Figure 6B, genes putatively involved in subsequent modification of chaetoglocin A were dramatically downregulated with CgPKS11 deletion, e.g., the mRNA level of a putative methyltransferase encoding gene CHGG_10646 as well as a putative cytochrome P450 family protein encoding gene CHGG_10649 significantly dropped to 21.6% and 35.9% of the wild-type level, respectively. However, the expression of CHGG_10650, a predicted transcription factor in the gene cluster, was not affected remarkably. This finding is significant and indicates that CgPKS11 may play a role in affecting the genes in the same biosynthetic cluster through some unclear pathway.

## 4. Discussion

Polyketides are the common secondary products of fungi. The widely spread fungus *C. globosum* is well studied for its capacity of making an abundant polyketide and is used as a biocontrol agent. One of the metabolites, ChA, strongly inhibits the egg hatching of root-knot nematode *M. incognita* [14] and has strong nematicidal activities against the second stage juveniles of *M. incognita* or *M. javanica* [14,27]. Considering the promising application of *C. globosum*, we intended to improve the yield of its main active product ChA by eliminating competing polyketide products in NK102.

Polyketide synthases (PKSs) are responsible for the biosynthesis of polyketide backbone. Usually, a single genome of filamentous fungi can harbor a number of PKS genes. For the majority, their biological function is largely uninvestigated. In the search for total PKSs in *C. globosum* by using bioinformatics tools, 28 putative PKS encoding genes were identified, which are located in 28 gene clusters with 24 type I PKSs, 3 PKS-NRPSs, and 1 type III PKS genes. A putative PKS with a gene locus CHGG_10647 was previously predicted to be responsible for the biosynthesis of the polyketide chaetoglocin A [20]. Here, we defined the domain structure of the putative protein product encoded by the homologous gene of CHGG_10647, designated as CgPKS11. As a rare type I PKS, however, CgPKS11 is without the ACP domain. The ACP domain is a small (~80–100 residues) and non-catalytic, either independent, freestanding structures, or covalently bound as part of multi-modular enzymes such as fatty acid synthases (FASs), PKSs, and NRPSs [28]. In known PKSs, ACP activates the acyl CoA substrates and channels the polyketide intermediates, except for type III PKSs that utilize acyl CoAs as substrates directly [29]. There is one ACP domain located in the key enzyme of ChA biosynthesis CgCheA [17]. CgCheA catalyzes the stepwise assembly of a nonaketide from an acetyl-CoA starter unit and eight malonyl-CoA extenders that are loaded onto the ACP domain by means of the acyl transferase (AT) domain. The product is then modified with a tryptophan and delivered to the PCP domain of CgCheA for subsequent steps. In contrast to the canonical loading strategy, a standalone ACP plays an important role in the biosynthesis of *β*-amino acid-containing macrolactams. The *β*-amino acid unit is first ligated with a standalone ACP VinL and then aminoacylated with L-alanine, prior to loading onto a modular PKS, in the biosynthesis of β-amino acid-containing macrolactams [30]. In some cases, the ACP domain of PKS is missing. In an analysis of PKS gene clusters in lichenizing fungi, six ACP-less PKS were observed in the genome of *Peltigera membranacea* and one ACP-less PKS was located in the genome of *Endocarpon pusillum* [31]. The reason for this unusual observation is still unknown. Some researchers think it is possible that these PKS were inactivated by evolution and have been rendered non-functional through loss of ACP domains to adjust its production to environmental conditions [31]. In research on macrotetrolide biosynthesis in *Streptomyces griseus*, the macrotetrolide type II PKS has been demonstrated to act non-iteratively, lacking of an ACP domain, to utilize acyl-CoA as substrate directly and catalyze both C–C and C–O bond formation [29]. CgPKS11 is an ACP-less type I PKS as well, although most of PKSs that share a high homolog with CgPKS11 usually contain an ACP domain (Figure 2B). We have searched for more than a dozen genes on both sides of the CgPKS11 gene cluster, but none where a predictable standalone ACP was located in the nearby loci. We further search for the potential standalone ACP in the genome of *C. globosum* CBS148.51 via Blastp. An ORF, CHGG_09364 was obtained. The ORF putatively encodes 1073 amino acids and is annotated as a hypothetical protein. The region covering 1006-1064AA of this protein contains a standalone ACP domain. However, it is silenced according to RNA-seq profiling (Table S3).

Secondary metabolites are often bioactive, usually of low molecular weight, and are produced as families of related compounds at restricted parts of the life cycle, with production often correlated with a specific stage of morphological differentiation [32]. We further investigated the function of this structurally unique PKS, CgPKS11, in several aspects, including the sexual development and ChA biosynthesis of *C. globosum*. We deleted *Cgpks11* via a CRISPR-Cas9 gene deletion system [22]. This is likely the first report of CRISPR-Cas9 system applied in *C. globosum* and will facilitate genetic engineering in this fungus. Our data revealed that the function of CgPKS11 participated in the sporulation process. Deletion of CgPKS11 resulted in delayed maturation of ascocarps, shortened cell length of ascoma hairs, and significant reduction of ascospores production. This is an unreported phenotypic consequence for a PKS in this fungus, although the mechanism of CgPKS11 on the fungal development is not clear. Substantial data demonstrated that PKS-derived melanin is required for the development of asexual and sexual structures of fungi that might function as protectants against harmful environmental conditions. Deficiency of PKSs responsible for pigment biosynthesis usually had a negative effect on sexual development [19,33,34,35]. Indeed, we previously demonstrated that downregulation of PKS-1/Alb1, a key enzyme responsible for pigment biosynthesis displayed a pigment-deficient phenotype and lost the ability to produce ascospores in *C. globosum* [19]. In contrast, deletion of CgPKS11 has no effect on morphological characteristics of ascospores, so CgPKS11 is unlikely to regulate sexual development through pigment biosynthesis.

Secondary metabolite production is also responsive to general environmental factors, like carbon and nitrogen sources, temperature, light, and pH. In some cases, polyketides or polyketide non-ribosomal peptide hybrid molecules, may act as signaling molecules to regulate development and physiology. Taking zearalenone as an example, which was an estrogenic mycotoxin synthesized by *Fusarium roseum*, it was reported to act as a sex-regulating hormone on perithecial formation [36]. Differentiation-inducing factor (DIF)-1 is a chlorinated alkyl phenone made from a polyketide and was reported to act as a chlorinated signal molecule regulating *Dictyostelium* development [37]. Nemamides, the hybrid polyketide non-ribosomal peptides produced by *Caenorhabditis elegans,* have been shown to act as regulators of starvation-induced larval arrest [38]. Studies on the function of FluP polyketide synthase in *Aspergillus flavus* suggested that FluP is involved in the synthesis of a diffusible metabolite that could serve as a signal molecule to regulate sclerotiogenesis [39]. Therefore, we believe chaetoglocin A, a small polyketide that is synthesized by CgPKS11, may act in a similar way. As deletion of CgPKS11, which is responsible for the biosynthesis of chaetoglocin A, led to slower growth of the mycelium and retarded sexual development, and in particular showed a dramatic rise of ChA biosynthesis. The yield of ChA from Δ*cgpks11* increased to nearly 1.6-fold of that from the WT strain. According to qRT-PCR analysis, we found that all genes of ChA biosynthesis gene cluster were simultaneously upregulated as expected. In addition, transcription of *pks-1* that previously reported to be involved in ChA biosynthesis was also upregulated. It explained why ChA yield was increased in Δ*cgpks11*. It should be noted that CgPKS11 deletion also led to the downregulation of the transcription of two other genes, CHGG_10646 and CHGG_10649, within the chaetoglocin A biosynthesis gene cluster. Taken together, the above studies suggest that either CgPKS11 or its product chaetoglocin A probably act as a regulation factor in sexual development and ChA biosynthesis.

## 5. Concluding Remarks

The molecular mechanism regulating ChA biosynthesis remains largely uninvestigated. We constructed CRISPR-Cas9 editing system for manipulation of the genome of *C. globosum* and attempted to improve ChA by a large leap and to study the control of its production at the molecular level. In the attempt to delete all the PKSs in order to eliminate the possible competitive pathways in the biosynthesis of ChA, a rare type I PKS, *Cgpks11*, which is responsible for the biosynthesis of chaetoglocin A and lacks the ACP domain, was deleted. The deletion of the gene resulted in the loss of chaetoglocin A, but astonishingly increased the production of ChA dramatically. In addition, CgPKS11 deletion led to growth retardation on agar plate and seriously impaired ascospore development, reduced the capacity of sporulation, and shortened ascoma hair cells. More importantly, we found that CgPKS11 modulated the ChA biosynthesis by inhibiting the expression of genes in the *CgcheA* cluster, which is responsible for ChA biosynthesis, while it downregulated genes for chaetoglocin A modification genes. Deletion of CgPKS11 released the inhibition. The findings revealed a novel mechanism taken by PKSs in coordination of growth, sporulation, and secondary metabolism in *C. globosum*. We propose that CgPKS11, probably through its product chaetoglocin A, and as a signal molecule, concerts secondary metabolism, growth, and sexual development in *C. globosum*. Hence, this work provides insights into the regulation of ChA biosynthesis and high-yield engineering construction and also demonstrates that PKSs are not only responsible for polyketide production, but also affect the growth and development of fungi. To construct stable ChA high-yielding engineering strains for ChA application, we perceive a future plan that should be done to uncover the precise crosstalk between ChA and chaetoglocin A biosynthesis, and to scrutinize on a larger scope of ChA biosynthesis in other fungi.

## Data Availability

All relevant data are available in the Supplementary Materials.

## Supplementary Materials

The following are available online at https://www.mdpi.com/article/10.3390/jof7090750/s1, Figure S1: Construction of *Cgpks11* deletion plasmid. Figure S2: Diagnostic PCR screening for *Cgpks11* deletion mutants. Figure S3: Southern blotting to confirm that Cas9 was eliminated in Δ*cgpks11* mutants. Figure S4: Effect of *Cgpks11* deletion on pigment biosynthesis during the liquid fermentation. Table S1: Putative PKSs presented in genomes of *C. globosum*. Table S2: Primers used in this study. Table S3: Expression of a predicted standalone ACP encoding gene CHGG_09364.
