# Supplementary material for: Deletion of a Rare Fungal PKS CgPKS11 Promotes Chaetoglobosin A Biosynthesis, Yet Defers the Growth and Development of Chaetomium globosum
Source: J Fungi (Basel). 2021 Sep 13;7(9):750. doi: 10.3390/jof7090750 (PMC8471558; doi:10.3390/jof7090750)
Supplement: Supplementary file 1 [file jof-07-00750-s001.zip › jof-1306046-supplementary.pdf]

Journal of Fungi

MDPI

Supplementary materials

Deletion of a Rare Fungal PKS CgPKS11 Promotes Chaetoglobosin A Biosynthesis, Yet Defers the Growth and Development of *Chaetomium globosum*

Biyun Xiang [1], Xiaoran Hao [2,*], Qiaohong Xie [1,3], Guangya Shen [1], Yanjie Liu [1] and Xudong Zhu [1,*]

[1] Beijing Key Laboratory of Genetic Engineering Drug and Biotechnology, College of Life Sciences, Beijing Normal University, Beijing 100875, China
[2] National Experimental Teaching Demonstrating Center, College of Life Sciences, Beijing Normal University, Beijing 100875, China
[3] Xiamen No.1 High School of Fujian, Xiamen 361000, China
* Correspondence: zhu11187@bnu.edu.cn (X.D.Z); 2015xrhao@bnu.edu.cn (X.R.H); Tel: +86-010-58804266 (X.D.Z); Tel: +86-010-58802159-812

ASSOCIATED CONTENT

Supplementary Tables and Figures

Table S1. Putative PKSs presented in genomes of *C. globosum*.

Table S2. Primers used in this study.

Table S3. Expression of a predicted standalone ACP encoding gene CHGG_09364.

Figure S1. Construction of *Cgpks11* deletion plasmid.

Figure S2. Diagnostic PCR screening for *Cgpks11* deletion mutants.

Figure S3. Southern blotting to confirm that Cas9 was eliminated in *Δcgpks11* mutants.

Figure S4. Effect of *Cgpks11* deletion on pigment biosynthesis during the liquid fermentation.

Table S1. Putative PKSs presented in genomes of *C. globosum.*

Gene in NK102	Gene in CBS148.51	Type of PKS	Location
Cgpks1	CHGG_00542	Type I PKS	Scaffold_1
Cgpks2	CHGG_03101	Type I PKS	Scaffold_2
Cgpks3	CHGG_05147	Type I PKS	Scaffold_4
Cgpks4	CHGG_09585	Type I PKS	Scaffold_7
Cgpks5	CHGG_04475	Type I PKS	Scaffold_4
Cgpks6	CHGG_08746	Type I PKS	Scaffold_6
Cgpks7	CHGG_05358	NRPS-Type I PKS	Scaffold_3
Cgpks8	CHGG_09586	Type I PKS	Scaffold_7
Cgpks9	CHGG_10092	Type I PKS	Scaffold_7
Cgpks10	CHGG_00046	Type I PKS	Scaffold_1
Cgpks11	**CHGG_10647**	**Type I PKS**	**Scaffold_7**
Cgpks12	CHGG_03589	Type I PKS	Scaffold_2
Cgpks13	CHGG_02374	Type I PKS	Scaffold_2
Cgpks14	CHGG_01021	Type I PKS	Scaffold_1

Cgpks15	CHGG_04068	Type I PKS	Scaffold_4
Cgpks16	CHGG_05286	NRPS-Type I PKS	Scaffold_3
Cgpks17	CHGG_05113	Type I PKS	Scaffold_4
Cgpks18	CHGG_10027	Type I PKS	Scaffold_7
Cgpks19	CHGG_10128	Type I PKS	Scaffold_7
Cgpks20	CHGG_07645	Type I PKS	Scaffold_5
Cgpks21	CHGG_01239	NRPS-Type I PKS	Scaffold_1
Cgpks22	CHGG_07638	Type I PKS	Scaffold_5
Cgpks23	CHGG_00087	Type I PKS	Scaffold_1
Cgpks24	CHGG_08147	Type I PKS	Scaffold_5
Cgpks25	CHGG_08793	Type I PKS	Scaffold_6
Cgpks26	CHGG_08141	Type I PKS	Scaffold_5
Cgpks27	CHGG_00246	Type I PKS	Scaffold_1
Cgpks28	CHGG_08934	Type III PKS	Scaffold_6

Table S2. Primers used in this study.

Primer name	Sequence (5′-3′)
act1p-F	**GCAGGCATGCAAGCT**CATCGTGATGGTCGTTGAAACC
act1p-R	**CTTCTTTGGGGCCAT**TTTGACGGCTGGAAAGGTGC
cas9in-F	ATGGCCCCAAAGAAGAAGCG
cas9in-R	**GGCCAGTGCCAAGCT**TCCCCAGCATGCCTGCTATT
CXcas9infu-1	ATCCGGTTCTTCCGTCTGGT
u6gdna1-F	**CGAAATTCGAGCTCG**CGATGCAATGCAGCTGGAA
u6gdna1-R	**CGGTCTTCATGAAGACTA**GAGGAAAGAAAGAAGAGA
u6gdna2-F	**TAGTCTTCATGAAGACCG**GTTTTAGAGCTAGAAATAGCAAGTT
u6gdna2-R	**CTAGAGGATCCCCGGCTCGAG**TAAAACAAAAAAGCAC
Hyg-Xba I-F	**TTTTGTTTTACTCGAGTCTAGA**GTCGACAGAAGATGA
Hyg-Xho I-R	**GGATCCCCGGCTCGAG**AAAGAAGGATTACCTCTAAACAAGT
PCgpks11-UF	**TTTACTCGAGTCTAGC**TACGAAACAAGCAGGATGGATGG
PCgpks11-UR	**TTCTGTCGACTCTAG**GCCTCACGGATACAATCTTCTTGG
PCgpks11-DF	**CCGGGGATCCTCTAG**GACACCGCTTTCTTTGAGTGCC
PCgpks11-DR	**TAGAGGATCCTCTAGA**GCGTGTAAGCAATATCAGCG
PCgpks11-N19-F	**CCTC**GATGCCGGTAACCTCCCCTT
PCgpks11-N19-R	**AAAC**AAGGGGAGGTTACCGGCATC
P11-KO-VP-F	ATAGGTTGTAGGTGGGCTG
P11-KO-VP-R	CGTTCTGGCTCATACTCAC
iHYG-R	GCAAAGTGCCGATAAACAT
PHYG-ter-F	TGAATGCTCCGTAACACC
HYG-F	CTAGAGTCGACAGAAGATGA
HYG-R	AAAGAAGGATTACCTCTAAACAAGT
qCgpks11-F	CGCTATCTACCTCAAGAAGTCA
qCgpks11-R	GAGGTTACCGGCATTTCTGT
q10646-F	CATTTCTCTGGCTGACCTTG
q10646-R	GACTTCATCTCTTCATCTCTGT
q10649-F	TCAAGCACCAATCTCCAGTT
q10649-R	GGCTTCTCAAAGTCGGTTC
q10650-F	GATACTTTGTCACCGAGGTC
q10650-R	CGAGGCGTAGCAACATATC
PCas9-PF	CCCACCATCTACCACCTGA

PCas9-PR	GCCGCCAGTAGTTCTTCAT
qGAPDH-F	AACGGCAAGAAGGTCAAG
qGAPDH-R	TCTCGGTGGTAGTGAACA
q01237-F	GGGAAGGACCGATACCATAAAC
q01237-R	TCTAAACCCCATTCTACAACCG
q01238-F	CAGAGGGATGTGGGTAAGGG
q01238-R	CTAACGTATATTATAAGCGAGCGA
q01239-F	GATTTGCCTCGGTTGTGCTTA
q01239-R	CATAGTGATACCTTGCGTTCTCC
q01240-F	GGTATTACAACGGATGCGACTT
q01240-R	CGGTAGGAGAACACGCTGAC
q01241-F	GAGGAATGGCACCAGGAAT
q01241-R	AGTTAAGCGGCAGCATCT
q01242-1-F	GGCTTCCAAAGCATACGCAG
q01242-1-R	TGGTGTTTGTTCGGGTCTCC
q01242-2-F	GCCGTGTCCGAGAGTTATGT
q01242-2-R	AGAGGAAAGCCAGCAGTTCG
q01243-F	CGGTCTTGCGGCTATTGAT
q01243-R	GCTGGCGACTTCTTGTCTG
q01244-F	CACACGCAACGAGTATATCCT
q01244-R	ATCGTGCTTTGCCGCTTC
q00542-F	ATCTTTCCGCCTAACCCGA [19]
q00542-R	GTCCTTCGTTTCTGGGTTGTC [19]

* The bold parts indicate the homologous arms of the primers. The underlined part represents the reserved restriction enzyme site.

Table S3. Expression of a predicted standalone ACP encoding gene CHGG_09364.

Gene symbol	Deduced function	FPKM_WT1	FPKM_WT2	FPKM_WT3	pval
CHGG_09364	ACP domain	1.53394	1.45815	1.37813	0.004947391

*FPKM: Fragments per Kilobase Million; WT1/WT2/WT3: Three culture replicates of wild-type strains have been used. pval: *p*-value.

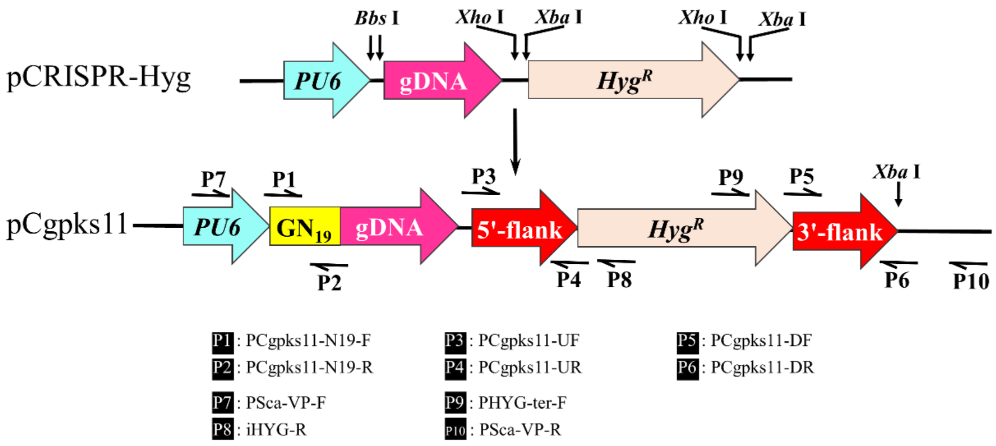

Figure S1. Construction of *Cgpks11* deletion plasmid. Primers for plasmid construction or diagnostic PCR are indicated in black boxes.

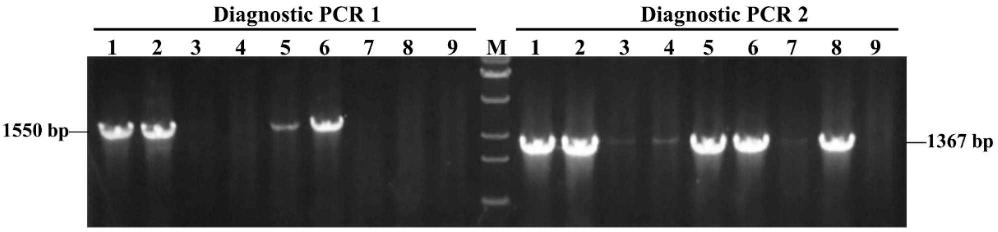

Figure S2. Diagnostic PCR screening for *Cgpks11* deletion mutants. A 1550 bp fragment could be amplified by primers P11-KO-VP-F/iHYG-R, and a 1367 bp fragment could be amplified by primers PHYG-ter-F/P11-KO-VP-R. Lanes 1-9 were loaded samples from transformants NO11-19. M: Trans15K DNA Marker.

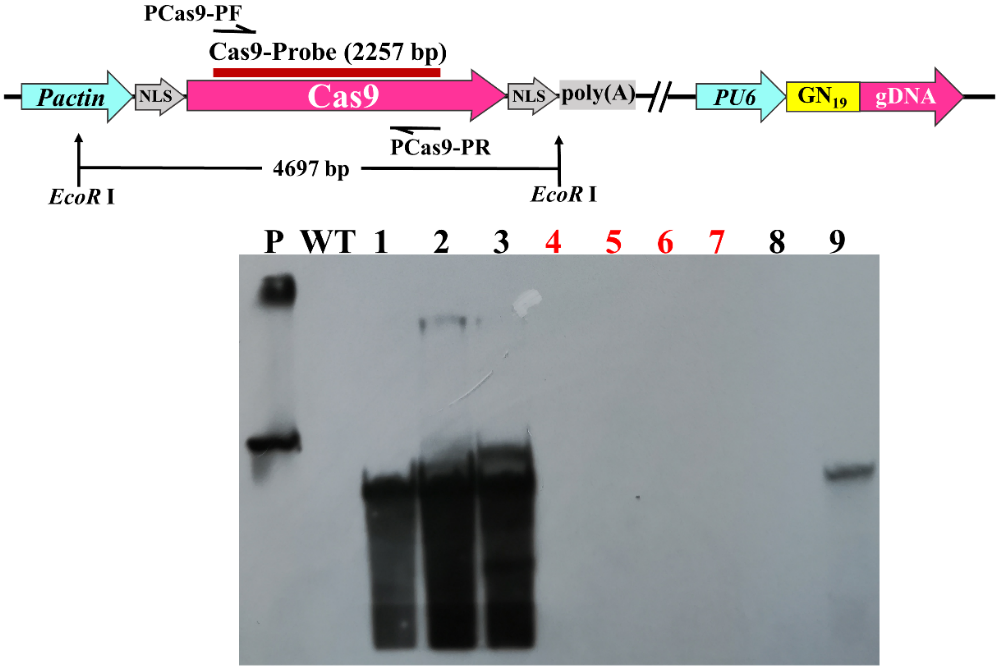

Figure S3. Southern blotting to confirm that Cas9 was eliminated in *Δcgpks11* mutants. Cas9-probe was labeled and used as the probe, the unlinearized Cas9-containing plasmid pCRISPR-Hyg was used as the control. Lanes 1-9 were loaded samples from randomly selected transformants NO1, NO3, NO9, NO11, NO12, NO15, NO16 NO23 and NO33.

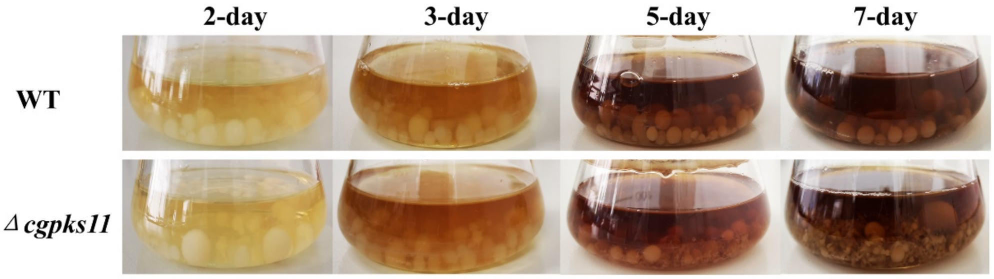

Figure S4. Effect of *Cgpks11* deletion on pigment biosynthesis during the liquid fermentation.
